# Vitalism in contemporary chiropractic: a help or a hinderance?

**DOI:** 10.1186/s12998-020-00307-8

**Published:** 2020-06-11

**Authors:** J. Keith Simpson, Kenneth J. Young

**Affiliations:** 1 College of Science, Health, Engineering and Education, Murdoch University, Perth, Western Australia; 2 School of Sport and Health Sciences, University of Central Lancashire, Preston, PR1 2HE UK

**Keywords:** Chiropractic, Vitalism, Social contract, Legitimacy, Cultural authority, Fiduciary duties

## Abstract

**Background:**

Chiropractic emerged in 1895 and was promoted as a viable health care substitute in direct competition with the medical profession. This was an era when there was a belief that one cause and one cure for all disease would be discovered. The chiropractic version was a theory that most diseases were caused by subluxated (slightly displaced) vertebrae interfering with “nerve vibrations” (a supernatural, vital force) and could be cured by adjusting (repositioning) vertebrae, thereby removing the interference with the body’s inherent capacity to heal. DD Palmer, the originator of chiropractic, established chiropractic based on vitalistic principles. Anecdotally, the authors have observed that many chiropractors who overtly claim to be “vitalists” cannot define the term. Therefore, we sought the origins of vitalism and to examine its effects on chiropractic today.

**Discussion:**

Vitalism arose out of human curiosity around the biggest questions: Where do we come from? What is life? For some, life was derived from an unknown and unknowable vital force. For others, a vital force was a placeholder, a piece of knowledge not yet grasped but attainable. Developments in science have demonstrated there is no longer a need to invoke vitalistic entities as either explanations or hypotheses for biological phenomena. Nevertheless, vitalism remains within chiropractic. In this examination of vitalism within chiropractic we explore the history of vitalism, vitalism within chiropractic and whether a vitalistic ideology is compatible with the legal and ethical requirements for registered health care professionals such as chiropractors.

**Conclusion:**

Vitalism has had many meanings throughout the centuries of recorded history. Though only vaguely defined by chiropractors, vitalism, as a representation of supernatural force and therefore an untestable hypothesis, sits at the heart of the divisions within chiropractic and acts as an impediment to chiropractic legitimacy, cultural authority and integration into mainstream health care.

## Background

It is an indisputable fact that the chiropractic profession is troubled by ideological divisions and hence lacks a clear identity. In simplest terms, one group of chiropractors are evidence-based musculoskeletal practitioners while the other ascribes either to the original vitalistic Palmerian ideology or a variant thereof. The World Federation of Chiropractic’s (WFC) Identity Consultation Task Force acknowledged “the fact that the chiropractic profession does not have, but urgently needs, a clear and effective public identity” [1] p.(i). The role of vitalism in the profession’s identity was explored by the WFC at its 2003 conference. A panel discussion entitled “Is Vitalism a Strong Foundation or Quicksand for the Chiropractic Profession” concluded that the core concept of vitalism is fundamental to chiropractic while recognizing that vitalism is a poorly understood term carrying considerable baggage [2].

The ongoing controversy about the identity of the profession leads to both intra and inter-professional conflict and societal confusion regarding chiropractic and stalled integration [1]. The World Health Organization and the National Institutes of Health in the USA identifies chiropractic as part of Complementary and Alternative Medicine (CAM) [3, 4]. Similarly, the Cochrane Collaboration includes chiropractic as part of CAM along with 50 other therapies ranging from aromatherapy to tui na, a form of Chinese manipulative therapy [3]. It seems however that the rank and file chiropractors see themselves differently. The majority (69%) of the chiropractors in a 2010 survey rejected being characterized as CAM practitioners preferring instead to be classified within Integrative Medicine (IM) [5]. IM combines the best of conventional western medicine and evidence-based CAM therapies and operates within mainstream health care [6]. Some chiropractic academics advocate that the profession should create an identity focussing on evidence-based spine care and conservative management of common musculoskeletal disorders [7] while others argue that vitalistic chiropractic could make a significant contribution as an alternative to conventional medical care in addressing the increasing burden of non-communicable diseases [8].

Despite these obstacles chiropractic has made extraordinary inroads into the health care system worldwide. Having emerged from the pre-scientific health care era in the United States of America (USA) in the early twentieth century it now has a global footprint with representation in approximately 100 countries. It is the third largest regulated primary contact health care profession in the western world [4]. Nearly two decades after Meeker and Haldeman published “*Chiropractic: A Profession at the Crossroads of Mainstream and Alternative Medicine* [9] chiropractic remains at the crossroads without what Nelson et al. described as an “understandable, credible and scientifically coherent” identity [7] p.1. Leboeuf-Yde et al. advised there is need to pause and consider the causes [10]. This paper is such a consideration. We contend that vitalism is at the core of the discord. We agree with Hawk’s 2003 caution at the 7th Biennial Congress of the World Federation of Chiropractic (WFC), held in Orlando, Florida: "an unthinking acceptance of vitalism is no different from an unthinking acceptance of the mechanistic model – they are both not only unproductive but actually obstrictive, since they perpetuate stereotypes and dogmatic inflexible thinking [2] p.7".

We sense there is a poor understanding of vitalism amongst those chiropractors adhering to a vitalistic ideology. Discussions with practitioners identifying as vitalistic typically have seen vitalism used the way Jennings outlined in 1913: “to signify merely the doctrine that mechanistic formulation is not adequate for giving an account of nature” [11] p.81. We assert that the profession needs to clearly understand vitalism before it can determine if it is an ideology worth retaining in the twenty-first century. It is our contention that with an understanding of vitalism will come recognition that it is a hindrance to professionalization and therefore an impediment to developing cultural authority. We believe that the path to a sustainable future for chiropractic lies in abandoning this discredited belief.

This paper has three aims. The first is to recount the history of vitalism. The second aim is to examine vitalism within chiropractic, historically as well as its current status. The third aim is to consider whether a vitalistic ideology is compatible with the legal and ethical requirements for registered health care providers such as chiropractors.

## Discussion

### History of vitalism

#### Vitalism defined

Vitalism is the doctrine that all living organisms are sustained by an inexplicable non-physical *vital force* [11–13]^1^ that is “both different from and greater than physical and chemical forces” [14] p587. This keystone belief leads to the common phrase within vitalism: the whole is greater than the sum of its parts. Vitalists contend that living matter is ontologically greater than the sum of its parts because of some life force added to or infused into the chemical parts [15]. The *vital force* is not only the source of life, but it is also a key component of health and healing.

Although the term vitalism did not appear before the nineteenth century [16], the doctrine itself is ancient. Hans Adolf Eduard Driesch, (1867–1941), the last great spokesman for vitalism, considered Aristotle to be “the first exponent of scientific vitalism” [13] p.11. Aristotle coined the word *entelechy* to identify whatever it is that makes the difference between mere matter and a living body. According to Driesch, the Aristotelian concept of life, with its emphasis on the activities of the soul, formed the cornerstone of all theorizing on biological matters well into the eighteenth century [13].

Driesch explained:

Entelechy is an agent *sui generis* [of its own kind; in a class by itself; unique], non-material and non-spatial, but acting " into" space, so to speak ; an agent, however, that belongs to nature in the purely logical sense in which we use this word [13]. p.204

The vital force or forces representing *entelechy* are culturally specific and have culturally specific names. In the Western tradition founded by Hippocrates, these vital forces were associated with the four temperaments and humours; in Eastern traditions *qi* or *prana* represent the vital forces; in Afro-Brazilian religions it is called *axé* [17]. Thus entelechy with roots traceable to Aristotle may be located through the *vis essentialis* of Wolff [18], to the entelechy of Hans Driesch [13] and the emergent *élan vital* of Henri Bergson [19] in the early twentieth century.

Coulter and Willis [14] described vitalism within health care using the term moderate vitalism. This they say is equivalent to *vis medicatrix naturae* or the healing power of nature [14]. This perspective views the practitioner as facilitating salutogenesis (the promotion of health within the body) [14], rather than counteracting pathogenesis (an attack on the body) [20].

To make a reasoned determination regarding the role of vitalism within health care, one must examine vitalism’s origins and meanings. This is no easy task because of vitalism’s heterogeneity and different connotative meanings [12, 21, 22].

Benton [23] proposed the following definition as the starting point in his in-depth examination of the topic.

Vitalism is the belief that forces, properties, powers, or ‘principles’ which are neither physical nor chemical are at work in, or are possessed by living organisms, and that any explanation of the distinctive features of living organisms which did not make reference to such properties, forces, powers or principles would be incomplete [23]. p.18

Benton acknowledges that while this definition is inclusive of the numerous vitalistic perspectives, it is so only because of its vagueness and ambiguity [23]. This is by no means a recent recognition, Lovejoy [12] in 1911 and Jennings [11] in 1913 drew their reader’s attention to the problems presented by attempting to determine what is meant by the term. As the twenty-first century approached, Lecourt suggested the term vitalism be abandoned altogether because of its ambiguity [24]. Notwithstanding the ambiguity, Benton’s definition holds favor in most discussions of vitalism.

Before exploring vitalism and associated explanatory models, it is worth briefly considering why humans have sought to describe why and how something works or strove to explain observed phenomena. Scottish philosopher David Hume held that humans have an instinctive belief in causality and that the concept of cause and effect, although faulty, is basic to human capacity to make sense of the world [25]. This, combined with an intrinsic human curiosity and need for analytical reasoning meant that over time humans have put forward biological theories to answer questions such as ‘what is life’ [26]. Creation myths and animism provided answers to such questions. Creation myths were developed to explain cosmic origins while animism and vitalism explained the difference between living organisms and non-living organisms [27].

### Explanatory theories: from creation myths to animism, Vitalism & Mechanism

#### Creation myths

In the pre-scientific era, creation myths, creation stories or cosmology myths explained how the universe and its inhabitants came to be [27]. They provided answers to key questions regarding our origins, geographical features and the existence of the cosmos. Creation myths are ubiquitous and ancient with most cultures believing the world was created by some purposeful action [28]. The Enuma Elish is considered the oldest written creation story, likely from the second millennium BCE by ancient Assyrians and Babylonians [29]. Creation stories were developed because the culture at that point lacked scientific explanations for existential questions. They were typically embedded in a culture’s religion and fulfilled an inherent human need to explain the physical world [30]. Characteristically, the stories are carefully preserved and transferred either in writing or orally across generations thereby becoming part of the culture [31]. Leeming and Leeming point out that creation myths describe an understanding significant to the culture and are retained whether or not advanced science exists [32].

Although creation myths provided answers to questions about how life originated, they did not address the question of what life actually is; what differentiates being from not being? This question is asking about human biology and physiology. It is apparent that animals, including humans, differ from inanimate objects such as rocks and vitalistic explanations were created to explain the difference [33]. It seems a logical corollary that if physical existence was purposefully created, so was life imbued to non-living tissue. This approach lends itself to the idea that the life force is unknowable or forbidden.

#### Animism

The earliest vitalistic explanation was likely animism: a theory of existence which held that all entities – plants, animals (including humans), inanimate objects and natural phenomena – possess a soul. It is a religious belief without an identified founder. Rather, it appears to have developed roughly simultaneously in various areas of the world [34]. While anthropologist Sir Edward Tylor was the first to document animism in his 1871 book, Primitive Cultures [35], scientists speculate that animistic beliefs go back to the earliest period of human development, the Paleolithic Period [36]. In simplest terms, animism, or the doctrine of the soul, is the belief that all things have a spirit or soul. This includes animals, natural phenomena (e.g. storms, floods, volcanos), plants, geographic features, and humans. All events are as the result of the initiation, coordination, and direction of an overseeing soul. Animism appears to be ubiquitous with forms identified in cultures in all regions of the globe including Americas, Africa, Asia, and Oceania. Tylor [37] explains that all humans have been concerned with two classes of biological questions:

First, what is it that makes the difference between a living body and a dead one; what causes waking, sleep, trance, disease, death?

Second, what are those human shapes which appear in dreams and visions? p392

The human soul or spirit was considered the cause of life and thought in the individual. It animated and possessed the personal consciousness of its corporeal owner and importantly, it is the departure of the soul or spirit that constituted death. Spirits could either be helpful or harmful to humans and thus spirits were worshiped or appeased. Animists offered sacrifices, prayers, dances, or other forms of devotions to these spirits in hopes of blessings upon areas of life (crops, health, fertility, etc.) or for protection from harm. While animism as a worldview has largely waned with the development of the great religions of the world, it continues to this day in some faiths for example in Japan’s traditional religion, Shinto [34, 38]. Animism is also evidenced today in some characterizations of inanimate forces (e.g. the fury of the tornado.) Even though animism waned, what remained was a belief that *something* distinguished a living creature, in particular humans, from inanimate matter and this *something* departed the body at the moment of death [26]. This basic vitalistic idea has taken root in many religions and cultures. Most familiar to the western world would probably be reference to the ‘soul’. Whatever the name, the *vital something* differentiating a living creature from inanimate matter was the incorporeal essence of a living being. Researchers agreed on many characteristics of the *something* however, they could not agree on the explanation for it. Consequently it is a mistake to presume that all vitalists are the same [39].

#### Fifty shades of vitalism

Volumes have been written on the typologies of vitalism with influential biologists championing each [23, 40]. Through all its iterations, vitalists retained certain elements such as the steadfast conviction to the distinctiveness of life and the operation of an irreducible vital principle in living beings. The following summary is drawn from DJ Nicholson’s PhD dissertation [41]. In it Nicholson presents a detailed analysis of the historical development of mechanistic and vitalistic conceptions of life since the seventeenth century [41]. Nicholson categorizes vitalists from the late 17th C to the present day into three categories: Animistic Vitalists, Somatic Vitalists and Naturalized Vitalists. Importantly for an understanding of the evolution of vitalism, Nicholson’s classification traces the ideology from its original construct [an unknowable unknown] to the more contemporary construct [a knowable unknown].

Animistic vitalists (AV) predominated from the late17^th^ Century to mid-eighteenth Century [41]. AVs accepted the *vital force* a priori*,* meaning it was simultaneously unknowable and forbidden, metaphysical and teleological [41]. It was a supernatural force, a gift from God or a god, which endowed the organism with life. GE Stahl was a leading philosopher in the AV typology. For Stahl the *vital force (soul)* involved an intrinsic purposiveness that could not be derived from merely physical-chemical forces [40]. The soul existed outside of living matter and its presence rendered the organism alive while its departure from the body resulted in the organism’s death.

In response to Newtonian natural philosophy by the mid-eighteenth Century, AV was superseded by what Nicholson calls somatic vitalism (SV). Somatic vitalists argued that the source of the *vital force* was not God-given, rather it resided within the organism although its source remained unknown. Caspar Friedrich Wolff was a key proponent of SV. To distinguish SV from animistic vitalism, Wolff referred to the *essential force* which fundamentally was the same as the *vital force* but not a gift from God. This shift away from a supernatural *vital force* source is exemplified by Wolff’s assertion: “This characteristic and essential force appears to be one [...] whose existence Stahl very certainly recognized, but which he, incorrectly I think, attributed to the soul” Wolff in Roe [42]. Wolff and other proponents of SV, such as Blumenbach and Driesch, agreed with Stahl that vital processes are inexplicable by mechanistic reductionism. The point of disagreement was in attributing these processes to the soul. The *vital force* was believed to come into being as the body forms during ontogeny and subsequently to reside in living matter. It was seen as a property of living matter upon which it supervenes. While this force could not be identified, its existence could be empirically confirmed through its observable effects on the organism. Somatic vitalism persisted until the early twentieth Century when it gradually gave way to what Nicholson calls Naturalized Vitalism (NV) [41].

Naturalized vitalists, while rejecting the notion of a life-soul force or some other unknowable force animating the organism, were still left with the question, what is the animating life force? For NV, the *vital force* was a heuristic device, a place holder and research hypothesis. It was a *vital force* of the gaps, something like a God of the gaps. In essence, vitalism and its associated vital force was a biological metaphor providing a basis for retaining our primary experience of life as a mystery while concurrently explaining the mystery through scientific analysis [43]. It was unknown at the current time, but the phenomenon or phenomena it represented would eventually be explicable by the principles of basic sciences like physics and chemistry, genetics, emergence and informatics [23, 26, 44–47]. This position was exemplified by French philosopher Bergson, a vitalist, who explained that vitalistic descriptions reminded us of our ignorance [48]. As research provided rational explanations, the understanding of biological and physical phenomena expanded and the need for vitalistic explanations diminished [16, 45, 49].

For example, Claude Bernard, a French physiologist and naturalized vitalist, was committed to finding the answers within the organism. Bernard’s concept of constancy and equilibrium in the *milieu intérieur* [the internal environment] was brought about by the organism’s capability to ‘self-regulate’ [a term used by Bernard]. Importantly for the ‘what is life’ discussion, Bernard was responsible for the adoption of physiochemical methodologies into biological investigation and thereby laid the foundations of modern biological research. This resulted in the development of the now accepted concept of homeostasis through the work of J. S. Haldane and Walter Bradford Cannon, two of the most prominent physiologists of the early-twentieth century. Homeostasis or dynamic equilibrium within the internal environment through continual compensatory adjustment was the term coined by Walter Bradford Cannon in 1926 [50] p. 24. It is important to note that homeostasis operates with no inexplicable *vital force* [51]. The work of the naturalized vitalists laid the foundation for the organicist movement that emerged between the First and Second World Wars and which has effectively rendered vitalism an historical construct [26].

#### Physiochemical mechanism [mechanism/physicalism] & Organicism

Mayr, one of the twentieth century’s leading evolutionary biologists, explains that the origins of mechanism are as ancient as those of vitalism [26]. The second section of Plato’s *Timaeus* depicts the Platonism cosmos as harmonious, mathematical and ordered. Plato was not alone in this portrayal with many others including Aristotle and Epicurus holding a similar view. These mechanistic views were largely lost or forgotten until the seventeenth Century with the beginning of the Scientific Revolution, which heralded the onset of a time when the authority of the ancients gave way to experimentation, exploration, testing, and research [52]. Within Descartes’ writings, he suggests a mechanical explanation of human physiology, including descriptions of sensation, respiration, muscle contraction, neurophysiology, digestion, reflex action, and the circulatory system [53, 54]. While there were many influences leading to a mechanistic worldview, René Descartes’ statement on the animal soul solidified mechanism as a worldview. Descartes claimed

Life, both human and animal, was a purely mechanical process and that the soul, which was absent in animals, did only that of which it was conscious, knew of what it thought and had no concern in vital activity [55]. p.227

Mechanism is a worldview describing the universe and its constituents as logically continuous and thus may be explained along physiochemical lines, thereby eliminating the need for reliance on supernatural explanations [56]. According to Marvin [56]:

All mechanists believe that whatever life may prove to be, no vital phenomena will be found to be inconsistent with physiochemistry; and the extreme mechanist believes that the phenomena both of life and of mind will in time prove to be fully explicable in terms of this logically prior science. p.618

Marvin’s differentiation between mechanists and ‘extreme’ mechanists alerts readers to the fact that mechanist had two meanings in the 19th and early 20th centuries. One group believed there are no supernatural forces at all, and the other group held there was no life-specific force(s) differentiating organisms and inanimate matter [26]. Responding to this differentiation, the term physicalism was introduced in 1931 by Neurath, a member of the influential Vienna Circle^2^ [57] to achieve clarity with a distinctively anti-mechanistic position that separates biological inquiry from theology and metaphysics [57]. Neurath presents physics as a unified science, from which theology and metaphysics have been swept away, a system of laws from which single events or processes are deduced [57] p.619,620. Physicalism posits that all things whether biological, psychological, moral, or social are either physical or supervene on the physical. The rapid development of physics - the study of matter, energy, and the interaction between them – saw mechanism evolve into the more sophisticated physicalism, however; while the terms mechanism and physicalism are often used interchangeably they should not be [58].

Throughout the nineteenth-century medieval vitalistic concepts were replaced by mechanistic ones. Some biologists espoused that all life could be explained in mechanical and physiochemical terms. However, explaining organisms from a mechanistic perspective was plagued with obvious limitations. Despite its ascendancy, Cartesian ideology [mechanism/physicalism] was adept at identifying parts and operations, it failed to provide an answer to what it takes for an organism to be alive [59]. Explaining life as a mechanism, reducing life to a system of levers, pulleys and physiochemical reactions, was unpalatable and importantly, could not explain the observation that the whole is greater than the sum of its parts. Worth noting also, such explanations conflicted with the ascendant Christian doctrine which effectively placed a religious filter on western natural philosophy [the study of the natural world, a pre-scientific state] in the middle ages [60]. While many advancements in knowledge and technology occurred, the Christian order established the conditions for the study of phenomena, with the focus being on the relationship between life and God’s laws [60].

Physicalists and mechanists attacked vitalists for citing *vital forces* to explain life while invoking ambiguous terms such as *energy* and *movements* into their explanations [26]. This led to a resurgent anti-mechanistic, vitalistic worldview and an ongoing debate between vitalists and mechanists/physicalists with both camps seeking to answer the question: what is life? Whether as a place holder or as an explanation, vitalism as a worldview appeared secure at least while there remained certain aspects of life unexplained by physiochemical mechanisms.

#### The decline of vitalism

The death of vitalism is sometimes attributed to Fredrich Whöler’s synthesis of urea in 1828 [61]. Wöhler synthesized an organic compound, from two inorganic compounds. Before Wöhler’s discovery, it was believed that organic compounds could be made only in plants or animals by a *vital force* that could not be replicated in the laboratory. Referred to by Ramberg [61] as the myth of Wöhler, it is an overreach to suggest that Wöhler’s discovery was a death blow to vitalism. As Warren [62] points out, Whöler himself doubted whether his discovery would have a negative impact on vitalism. It is safe to say, however, that the synthetic generation of an organic compound from two inorganic compounds dealt a serious blow to the vitalist hypothesis [61, 63, 64].

According to Mayr, several events contributed to the decline of vitalism, with the two most important being the rise of Darwinism and genetics in the latter half of the nineteenth century [26]. Darwin made at least nine major claims in direct conflict with the prevailing vitalistic religious beliefs [65]. His ideas sparked an ideological revolution both in the world of biology and within the average person’s world view [65]. Farlow wrote that Darwin’s 1859 Origin of Species “fell like a bomb in the [vitalist] camp and shattered time-worn theories” [66] p.80 which ultimately changed biology in the United States and elsewhere [67].

The study of genetics began in the nineteenth century with Gregor Mendel, who discovered the fundamental laws of inheritance [68]. Mendel’s work was presented at meetings of the Natural History Society in 1865. It laid the foundation for modern genetics, and genomics [69] effectively driving another nail into vitalism’s coffin. The impact of genetics on modern health care has been profound. For example, genome sequencing has a significant impact on stratifying cancer, characterizing genetic disease, drug prescription and development and providing information about an individual’s likely response to treatment [70].

The vitalists were not mortally wounded by Darwinism and genetics. Rather, what emerged about 1840 and enduring until the mid-1920s was neovitalism [71, 72] because, as Sumner noted, “vitalism will not down” [73] p.103. Sumner explained:

The strength of vitalism's appeal has a twofold basis: (1) the manifest failure of dogmatic mechanism, as thus far formulated, to explain (even in the sense of adequately describing) certain conspicuous facts of development, function, and behavior; and (2) the unpalatable corollaries, religious and ethical, which are supposed to follow inevitably the acceptance of a radical mechanism [73]. p.103

#### Neovitalism

Hans Driesch was the most prominent neovitalist. In his seminal work, *The History and Theory of Vitalism,* he derogated Darwinism as uselessly destructive and not a useful contribution to the debate, stating that Darwin “explained how by throwing stones one could build houses of a typical style” [13] p.137. Driesch further asserted that neovitalism emerged as an anti-Darwinian theory response [13] p.138. Like original vitalism, neovitalism’s central ideology was that life is unique, with an animating life-principle [71, 74]. Life is irreducible to physicochemical analysis and only partially conducive to laboratory experimentation but the life-principle is not itself of material nature [75]. Neovitalists fought against a physiochemical mechanist ideology with writing rich in metaphysical rhetoric [76]. But in something of a concession to mechanists, Dreisch insisted that the vital principle has absolutely no existence independent of physico-chemical matter [75]. This clearly positioned neovitalists into Nicholson’s somatic vitalist category [41] while placing neovitalists at odds with contemporary biological scientists because of the insistence that an unknown agent obstructed biological understanding [77].

Because neovitalism brought nothing new to the debate, critics were quick to comment. Welby asserted that neither vitalism nor neo-vitalism had anything to offer modern biological inquiry [71]. MacDougall [74] reminded readers that neovitalism was an adulteration of the scientific method. He acknowledged that science had not uncovered all the answers, but to invoke vitalistic entities as explanatory hypotheses was an unacceptable misunderstanding of the logic of science [74]. Johnstone, an English biologist, was less tactful when he advised that neo-vitalism was a “recrudescence of vitalism” exhibiting a “crude and even grotesque spiritualism” [72] p.13 while pioneer geneticist Frances Crick advised that “neo-vitalists [are those] who hold vitalistic ideas but do not want to be called a vitalist” [78] p.22. Needham, a leading chemical embryologist offered a less caustic assessment of neovitalism:

The neo-vitalist school, which some years ago seemed to have the future in its own hands, has not been able to live up to its promises. The more its special entities immanent in living beings have been considered, the less necessary they have seemed to be in relation to the facts and the less valuable they have been found as inspirations to research [79]. p.87-88

The rise of Darwinism and genetics “succeeded in providing valid interpretations of the phenomena claimed by vitalists not to be explicable except by invoking a vital substance or force” [26] p.14. This meant by the twentieth century vitalism and its first cousin, neovitalism, no longer served a purpose other than as a biological metaphor for naturalized vitalism. These developments lead physiologist J. S. Haldane in 1931 to declare “Vitalism came, however, during the latter half of last century, to be almost universally rejected by biologists, and for a very good reason” [80] p.9.

Two problems remained. The first was failure by vitalists to recognize vitalism as a biological metaphor, instead espousing the ideology as dogma [81]. The second problem was that neither vitalism nor physiochemical mechanism offered a true understanding of the intricacies of living organisms [26]. The former was rejected on scientific grounds because any hypothesis involving vitalistic concepts was untestable by scientific means. But the latter, while essential to understanding, could not explain the observed coordination so characteristic of life. It could only describe its component parts. A new theory of living matter going beyond vitalism and physiochemical mechanism was needed [82]. In 1919 WE Ritter, an American biologist, successfully argued that organicism [materialistic holism] provided the model required.

#### Organicism, systems biology and synthetic biology

Organicism incorporates several interrelated ideas:

- physiochemical mechanism is inadequate when applied to biological organisms;
- the whole is greater than the sum of the parts;
- the parts cannot be understood when considered in isolation from the whole;
- the parts are interdependent;
- interdependence of the parts results in emergent properties that are not apparent when considering the parts in isolation [15, 83, 84].

This approach, when combined with discovering the structure and function of genetic material, giving consideration to the thermodynamic aspects of living organisms, and employing bioinformatics has seen biology evolve into what is now known as systems biology [85]. Systems biology studies complex natural biological systems as integrated wholes, using tools of modeling, simulation, and comparison to experimental findings. Closely related to systems biology is synthetic biology which seeks to build artificial biological systems using many of the same tools and experimental techniques [86]. Both systems and synthetic biologists share the belief that organisms are made of partially independent functional modules organized in networks and recognize that biological systems have emergent properties that only a system considered as a whole can have [87]. These properties are not found in its component parts. From a mechanistic, reductionist ontology the characteristics of models that explain the behavior of the system are linearity, predictability, and determinism. In contrast, a systems ontology insists on non-linearity, sensitivity to initial conditions, stochasticity and chaotic behavior [88]. A systems-oriented overview of the concept of health implies robustness, adaptability/plasticity, and homeostasis [89] all without invoking any nonmaterialist claim or magical thinking.

Morange [87] argues that systems biology and synthetic biology represent the final step in providing natural explanations for biological phenomena and “to weed out teleological explanations” p.551. He suggested that the best way to eliminate the mystery from organisms would be to synthesize a living organism ‘from scratch’ from inorganic and organic components [87]. Such an achievement is no longer a matter for science fiction. In 2016 a group of researchers designed and synthesized a bacterial genome. For the first time synthetic DNA has been in complete control of a cell [90]. The answer to the question ‘what is life’ no longer contains a mysterious *vital force*. To wit, beyond a metaphor, vitalism no longer serves any purpose in biology. Health and health care is an amalgam of physiology, biology, psychology and sociology [91]. Given that vitalism no longer serves any purpose within biology, it is a corollary that vitalism no longer serves any purpose within health care. However, what role does vitalism play within chiropractic?

#### Vitalism within chiropractic

Using spinal manipulative therapy (SMT) as part of a health care regime is both ancient and widespread. The history of SMT in Europe dates to 400 BCE and a similar length of time in Mesoamerica [92, 93]. It can be traced as far back as 5000 years worldwide with representation in Chinese, Egyptian, Greek and Roman medical practices and traditions [94]. The more contemporary understanding of SMT in health care began with osteopathy and chiropractic emerging in the USA in the late 1800s [92]. At this time spinal irritation was believed to cause a host of afflictions ranging from nausea, vomiting, to upper and lower gastrointestinal tract symptoms, and reproductive tract symptoms [95]. Even as spinal irritation was falling into disfavor as a diagnosis [96, 97] the construct was embraced by DD Palmer. Known as The Discoverer of chiropractic and The Founder of the chiropractic profession [98], DD Palmer combined spinal irritation with a form of neurocentric vitalism (NV) [99] to formulate what Folk [100] labeled “vertebral vitalism” p.3. Neurocentric vitalism emerged in the mid-seventeenth century in conjunction with the neurocentric interpretation of medicine which began with Willis’ exploration of brain anatomy and vitalism represented a move away from animistic vitalism toward somatic vitalism [99]. Willis’ work was responsible for the neurocentric focus within medicine which continued into the latter half of the eighteenth century and likely played a role in Palmer’s emphasis on the nervous system as a key to health care [101]. However, Palmer’s vertebral vitalism was a pseudo-move from animistic vitalism because Palmer advocated that the vital force was God-given.

It is well established that DD Palmer’s (DD) ideology was a blend of vitalism and science with strong religious overtones [102]. He believed he had answered the question being asked by natural philosophers for centuries: What is life? [103] p.1. DD wrote of Universal and Innate Intelligence which were animistic vitalistic constructs linked to a Divine being and responsible for everything in the universe, including life. Palmer [103] penned:

Innate is part of the all-wise. Innate is a part of the Creator. Innate spirit is a part of Universal Intelligence, individualized and personified. p. 691

God - the Universal Intelligence- the Life-Force of Creation. p. 446

DD’s animistic vitalistic ideology was adopted and adapted by chiropractic ‘philosophers’, primarily BJ Palmer, and Ralph W Stephenson. Stephenson laid out 33 principles of chiropractic in his 1927 Chiropractic Textbook which enshrined chiropractic vitalism in the first two principles. The vitalistic cause of disease is set out in principles 30 and 31.

1. The Major Premise – A Universal Intelligence is in all matter and continually gives to it all its properties and actions, thus maintaining it in existence.

2. The Chiropractic Meaning of Life – The expression of this intelligence through matter is the Chiropractic meaning of life.

30. The Causes of Dis-ease – Interference with the transmission of Innate forces causes incoordination or dis-ease.

31. Subluxations – Interference with transmission [of Innate Intelligence] in the body is always directly or indirectly due to subluxations in the spinal column [104]. p.xxxi, xxxviii

By way of clarification, Stephenson informs readers:

The Science of Chiropractic holds that a Universal Intelligence created and is maintaining everything in the universe. This is manifested by movement and is called Life. A specific, definite portion of this intelligence, localized in a definite portion of matter and keeping it actively organized, is called by Chiropractic, Innate Intelligence [104]. p.1

BJ Palmer, the Developer of Chiropractic, left no room for doubt: by getting in touch with one’s own Innate Intelligence, one could contact God directly [105].

Clearly, these influential founding fathers all considered that a *vital life force* was central to health and disease and that subluxations interfered with this life force. According to chiropractic vitalists chiropractic’s contribution to health care is the location and removal of subluxations thus removing interference with the flow of Innate Intelligence [106, 107].

In its infancy chiropractic was promoted as a viable health care substitute in direct competition with the medical profession. Palmer declared in his subluxation theory that most diseases could be cured by adjusting [manipulating] subluxated vertebrae thereby removing interference with nerve vibrations flowing from the brain to the spinal cord and out through openings between the vertebrae [108]. So long as there was no interference a healthy functional state could be maintained.

In part because of its vitalistic ideology, chiropractic came into direct conflict with the emergent dominant health profession, orthodox [scientific] medicine [109, 110]. The result was that for the first seven decades chiropractors were considered deviant interlopers within health care and the profession was a marginalized outcast [9, 111, 112] which needed to be “contained and eliminated” as a health hazard in the United States [113] and elsewhere [114]. Chiropractic was attacked and criticized by political medicine on two fronts. The first was that Palmerian ideology was pseudoscientific and the second was that chiropractic lacked scientific legitimacy [115]. Chiropractic survived largely because of its clinical legitimacy – the continued patronage of clients willing to pay for chiropractic services [110, 116]. Now, in the twenty-first century, vitalism has been discredited and abandoned as an ideology within mainstream health care, but it remains an integral part of the culture of chiropractic.

DD’s concept of Innate Intelligence has been the subject of much criticism within the profession [107, 117–121] to the point of Bryner, in 1987, advocating abandonment of the term altogether because of its unscientific, dogmatic-sounding connotations [122]. This led DeGiacomo to acknowledge that Innate Intelligence is “perhaps the most controversial phrase in chiropractic” [123] p10. Possibly to deflect criticism and make vitalistic chiropractic more acceptable to mainstream health care, in 2005 Jolliot suggested something of a sleight of hand by having chiropractors instead use the word vitalism which is code for Innate Intelligence [124]. It is worthwhile noting that some chiropractors consider Innate Intelligence to be synonymous with homeostasis [121] however, homeostasis operates without the intervention of a *vital force* and consequently is not equivalent to Innate Intelligence.

Even as vitalistic thinking was rejected amongst mainstream scientists and health care providers in the early twentieth century [55, 125], for many within the chiropractic profession a subluxation-based paradigm rooted in vitalism became and remains their reality [126]. In 1996, David Koch, Senior Vice President of Sherman College of Straight Chiropractic examined the question of vitalism in chiropractic being an asset or liability. Koch asserted “a vitalistic view of the nature of life has been not only a help but also a fundamental necessity to the science and art of chiropractic” [127] p2. In 2010 Howard Vernon, a professor at Canadian Memorial Chiropractic College (CMCC), suggested that the profession’s subluxation theory has transformed from a vitalistic one to a mechanistic or physiologically-based understanding [128]. According to Vernon, this evolution “allowed many chiropractors to leave their vitalistic heritage behind to its rightful place in the history of ideas and move into a solidly, if not fully, mature physiologic/pathophysiologic model” [128] p. 24. Vernon’s suggestion appears to only be partially correct.

In 2010 the British General Chiropractic Council (GCC) issued ‘Guidance on Claims Made for the Chiropractic Vertebral Subluxation Complex’ [129] which directed practitioners that claims that the vertebral subluxation complex caused disease are unsubstantiated and must not be made. The GCC updated the statement in 2017. In 2014 the WFC formulated an education position statement supporting evidence-based chiropractic. In doing so the WFC distanced itself from vitalistic chiropractic [130]. Based on the WFC’s education position statement, the International Chiropractic Education Collaboration (ICEC), representing 12 teaching institutions worldwide, published a document entitled ‘Clinical and Professional Chiropractic Education: A Position Statement’. The ICEC Position Statement endorsed chiropractic education based on evidence-based care and renounced vitalistic subluxation as anything other than an historical construct [131]. Early in 2019 CMCC became a signatory to the ICEC’s updated position statement updated to include endorsement of the World Health Organization’s ‘WHO’s vision and mission in immunization and vaccines (2015–2030) [132].

These moves sparked a prompt reaction from The Chronicle of Chiropractic (Chronicle) which identifies as “the source of reporting on conservative, traditional chiropractic” [133]. Entitled “Canadian Memorial Chiropractic College Joins Anti-Subluxation Hate Group”, the Chronicle’s report of CMCC’s signing informs readers

The Position Statement falls in line with similar statements and the overall movement among the Chiropractic Cartel and the Subluxation Deniers that run it. Such positions have taken root in the United Kingdom, Australia, Canada and in some areas of the United States [133].

The Chronicle of Chiropractic is not alone in its support of vitalistic chiropractic. Hawk [134] advocated in her 2005 paper that maintaining a vitalistic perspective is appropriate in the post-modern era where there is no absolute version of reality, no absolute truths. Hawk [134] concludes

Vitalism, approached in a responsible and intelligent manner, may afford the chiropractic profession opportunities to further improve patient care and make contributions to new knowledge. p2.

Callender argues for a vitalistic chiropractic paradigm because it is a good fit with contemporary general systems theories [135]. Well into the twenty-first century Hart suggests the profession’s identity should be in line with the vitalistic founding principle of analysis and adjustment of vertebral subluxation to enhance wellbeing [136]. Similarly, Richards, Emmanuel and Grace posit that vitalistic chiropractic is a viable replacement for what they argue is a failing biopsychosocial model of health care [8] as does Russel [137]. Educationally, Life University College of Chiropractic’s (LUCC) strategic initiative is to “Become the preeminent performance-centered, vitalistic health care institution in the world” [138] p.IV. LUCC aims “be the visible leader in the academic discussion of vitalistic health care” [138] p.IV. The sentiment expressed by LUCC is not isolated. The Australian Chiropractic College (ACC) Initiative [139] is a non-university-based private college proposed by a group of vitalistic chiropractors, who adhere to the Palmerian subluxation theory. The ACC is well advanced in its plans to teach vitalistic chiropractic in Australia with its first open day in November 2019 and student intake in 2020 [139]. For those wanting to ‘immerse themselves in all things vitalistic and chiropractic’ the In8 summit is the biggest Vitalistic Chiropractic seminar in Australasia [140].

Organizationally, the Rubicon Group (RG) [141] and the International Chiropractors Association (ICA) [142] embody vitalistic chiropractic. RG is a world-wide collaboration of chiropractic educational institutions which embrace a vitalistic ideology. It emphasizes the significant role of what they have branded as “neo-vitalism” within chiropractic. This is characterized as neurologically-centered, subluxation-oriented approach to chiropractic and the group promotes vitalistic chiropractic education worldwide [143]. The ICA, the professional association founded by BJ Palmer, has as its main objective to

Maintain and promote chiropractic’s unique identity as a vitalistic non-therapeutic, non-allopathic, drugless and surgical-free health science, based on its fundamental principles and philosophy”

and its Mission is

To protect and promote chiropractic throughout the world as a distinct health care profession predicated upon its unique philosophy, science, and art of subluxation detection and correction.

[142]

Based on these examples there is no sign of vitalistic ideology dwindling within chiropractic. In fact, use of the term subluxation in chiropractic teaching facilities is widespread and growing, particularly in the USA [144]. It is readily apparent that for adherents, the chiropractic vertebral subluxation is a vitalistic construct with a purported impact on human function and health. Conversely, there is a large movement within the profession that recognizes vitalistic chiropractic as an historical entity. Clearly there is a schism in the profession with one group dis-endorsing vitalistic chiropractic with another group vehemently defending it. This raises the question: does retention of a vitalistic ideology have a positive or negative impact on chiropractic identity and legitimation?

### Vitalism and chiropractic: implications for the profession

We have examined the history of vitalism and vitalism within chiropractic. Now we will consider whether a vitalistic ideology is compatible with the legal and ethical requirements for registered health care professionals (HCP) such as chiropractors. This will be accomplished first by examining the concept of a profession and the social contract between a health care provider and society. We will then discuss the legitimation of chiropractic, its integration of chiropractic into mainstream health care and the effect that vitalism has on that process.

#### What is a profession?

The earliest recorded use of the word profession was by Scribonius Largus, a physician in the court of the Roman Emperor Claudius in 47 CE [145]. Scribonius referred to the “profession” of medicine, which he defined as a commitment to the relief of suffering [145]. Using the word profession to describe or define an occupational group came much later with the emergence of the ‘Learned’ professions – Theology, Law (canon and civil or common) and Medicine – in the late 1700s. The word profession is derived from the Latin: professionem meaning public declaration or oath. Members of the learned professions formed professional guilds which were similar in function to the craft guilds because they were strong, self-governing fraternities with entry by examination, expert training, with behaviours prescribed by a code of ethics, the enforcement of which remained the domain of the profession [146].

Eliot Freidson was a leading theorist about professionalization, particularly of medicine [147]. Freidson advised writers to first clarify what they mean by the term profession before conferring the title on an occupational group [148]. The concept of a profession involves a blend of specialized knowledge, jurisdictional control and societal recognition [149, 150]. Sociology emerged as a discipline in the late nineteenth century and sociologists and educators began studying the professions in the early twentieth century [151]. Several frameworks have emerged for the classification of the division of labor into occupations and professions. Talcott Parsons took a functionalist approach which was highlighted in his 1939 essay [152]. Parsons’ emphasizes that a professional embraces rationality while altruistically performing services to their clients, patients or pursuing impersonal values such as scientific advancement [152]. George Ritzer, a mid-twentieth century sociologist developed a continuum on which all occupations could be placed. It ranged from the non-professions on one end to the “established professions,” on the other [153] p49. An occupation’s position on the continuum is determined by the number of professinoal characteristics displayed as well as the degree to which the occupation possesses those characteristics. Those at the higher end (e.g. medicine, law) displayed 'professional attributes' while those at the lower end (e.g. draftsmen) displayed few or none.  Ritzer [153] detailed six professional attributes:

1. General systemic knowledge. Each profession has a unique body of knowledge which is acquired formally through prolonged training in a professional school and informally through professional relationships. p.56
2. Authority over clients. There are two aspects to this characteristic. First, the existence of a clearly defined client. Second, a large degree of authority over the client due to the relative lack of client expertise to determine what their needs are. p. 57
3. Community rather than self-interest. p.59
4. Internal rather than external control. This refers to professional autonomy to control entry into the profession as well as professional training and discipline. p. 60
5. Societal and legislative recognition of the profession. These is of particular importance because the profession secures monopoly within their area of expertise. p.61
6. A distinctive occupational culture which includes norms, values and symbols. p.63

Ritzer also notes that it is likely there will be conflict within the group in resistance to professionalization [153] p.63.

The problem was that by the late twentieth century a host of occupational groups were claiming professional status with its associated monopoly and autonomy at the expense of altruism and excellence [154, 155]. Current lists of professions include everything from animal trainer to disc jockey and zookeeper [156]. Within health care however, the attribute of altruism is enshrined in the special relationship between practitioner and client – the fiduciary relationship. According to Sharpe, the fiduciary relationship is based on dependence, reliance, discretionary authority and trust [157]. In health care, a vulnerable client is in a position of what is at times, extreme dependence on the expertise of the care provider and by definition the relationship is fiduciary [158–161]. The HCP has duties of care, loyalty and disclosure, which form the framework for the moral and legal responsibilities of the HCP to client [162]. In recognition of this powerful relationship and the responsibilities inherent therein, Elliott suggested that the term status profession be reserved for those occupational groups with the recognized foundational elements and having a fiduciary relationship with their clients [163]. Status professions include lawyers, accountants, police, clergy, teachers and registered health care professionals (HCP).

#### The contract between status professionals and society

This relationship between the status professional and their client is based on the social contract, a term introduced by Jean-Jacques Rousseau in 1762 CE [164]. Rousseau’s work was influenced by the social theories of Thomas Hobbes and John Locke [165]. Simply stated the social contract is an implicit collaborative agreement between members of a society outlining necessary relationships between individuals and their government for social benefits [166]. In 1982, sociologist Paul Starr used the term social contract to describe medicine’s relationship to society [109]. Since then there is wide agreement the social contract model is the best way to describe the relationship between HCPs and society [146, 167–170]. Cruess and Cruess, medical practitioners at McGill University in Canada, have published extensively on the social contract with the medical profession [171–173]. Parties to the social contract in health care are the profession and society, each with a set of expectations and obligations [173]. As Cruess and Cruess explain [173], because of the specialized knowledge of the profession, they are given a virtual monopoly over its dissemination and application with varying degrees of autonomy over governance of the profession. It incorporates the concept that professionals have duties above and beyond those of the average citizen. Cruess and Cruess [173] emphasize that the specialized nature of the knowledge plus a commitment to altruism supports society’s granting autonomy to the profession for establishing and maintaining the highest standards of practice for their calling. There is societal expectation that the professionals will strive for excellence by ongoing expansion of their knowledge base through research to ensure the highest standards of care. The contract contains implicit and explicit components underpinned by professionalism. The explicit aspects are within codes of conduct and practice standards which detail the legal obligations on the part of the health care profession and its members. The implicit components are embodied in the expectations of both the profession and society including the moral commitments expected of professionals. All parties have an obligation to fulfil the other’s legitimate expectations (Table 1). It is clear that societal expectations are extensive compared to those of the profession. This reflects the power imbalance within the fiduciary relationship. The fiduciary (the doctor) is the stronger party due to their specialized knowledge while the fiducie (the person whose good is held in trust by the fiduciary [174]) is the weaker, vulnerable and dependent party with resultant higher societal expectations [173].

**Table 1:** Societal and Health Care Professional (HCP) Expectations Under the Social (Fiduciary) Contract Adapted from Cruess and Cruess, 2008 [173])

Societal expectations:	The HCP will provide altruistic service to and support for their clients and society.
	The HCP will act with honesty and integrity with a moral commitment to the ethic of medical service.
	The HCP will provide competent, caring, compassionate high-quality service.
	The HCP will respect patient client dignity, autonomy and confidentiality.
	The HCP will comply with regulatory provisions.
	The HCP will have input into and support for health policy & public health initiatives.
	HCPs will cooperate and collaborate in client care.
Health care Professional (HCP) expectations:	The profession has sufficient professional autonomy to self-regulate.
	The profession has monopoly over its domain through licensing laws.
	The profession receives societal status in the form of respect, societal trust, and cultural authority.

The social contract is by no means constant. Over the last decades of the twentieth century and into the twenty-first century, professional autonomy has been eroded as societal trust and confidence in the professions waned even though society recognizes the services of status professionals as essential to its well-being [169, 175, 176]. Sullivan [175] explains that only fulfilling their obligations under the social contract will prevent further erosion professional autonomy. He advises status health care professionals to “strengthen and extend the kind of fiduciary morality that has long been part of the ethos of medicine” [175] p.675. With the social contract in mind and reflecting Sullivan’s advice, Cruess, Johnston and Cruess [177] developed a definition of profession for medical educators. This is the definition we used in our analysis of vitalism and the chiropractic profession.

An occupation whose core element is work based upon the mastery of a complex body of knowledge and skills. It is a vocation in which knowledge of some department of science or learning or the practice of an art founded upon it is used in the service of others. Its members are governed by codes of ethics and profess a commitment to competence, integrity and morality, altruism, and the promotion of the public good within their domain. These commitments form the basis of a social contract between a profession and society, which in return grants the profession a monopoly over the use of its knowledge base, the right to considerable autonomy in practice and the privilege of self-regulation. Professions and their members are accountable to those served and to society [177]. p.74

### The effect of vitalism on the chiropractic Profession’s social contract

#### Vitalistic subluxation-based chiropractic: ethical considerations

Stephen Perle, a chiropractor who teaches and writes in ethics, analyzed subluxation-based chiropractic through the lens of descriptive ethics, metaethics and normative ethics. Because of the lack of evidence that vertebral subluxations are detrimental to one’s health, he concluded that vertebral subluxation-based chiropractic breaches the duty of fidelity and is likely a violation of the practitioner’s nonmaleficence duty [178]. Furthermore, promotion of subluxation-based care frequently employs fallacious use of appeal to fear techniques rendering practitioners open to allegations of misleading and deceptive conduct or unconscionable conduct [179].

Viewed another way, based on these analyses vertebral subluxation-based chiropractic violates fiduciary duties and thus breaches the contract chiropractic has with society.

#### The effect of vitalism on the legitimacy of the chiropractic profession

Chiropractic has achieved many of the status professional traits outlined above but is lacking in critical areas as witnessed by its continued CAM status. As such chiropractic would be considered an emerging profession [180] and consequently lacking legitimacy. Max Weber, a founding father of sociology, introduced the concept of legitimacy and legitimation. Legitimation is a politico-legal process whereby a group gains legitimacy which Weber described as power or authority over goods or services as well as popular acceptance and recognition by the public of the authority [181]. As applied to professions, this would be monopoly over a professional domain. Willis advises that with legitimacy comes many benefits for the profession – social, political and financial – and, in Willis’ words “represents a place in the sun for its practitioners” [182] p.59. We will now examine the effects of vitalism on the legitimacy of chiropractic.

Medical anthropologist Ann Cobb [183] noted parallels between Ayurvedic medicine and chiropractic in the USA. Like Ayurveda, chiropractic established some elements of professionalization and legitimation, such as teaching institutions, professional chiropractic associations, research institutes, post-graduate seminars, chiropractic journals, and unique diagnostic tools. But Cobb found that examination of these characteristics beyond the superficial revealed them to be spurious for chiropractic. The teaching institutions in the USA are mostly private, run as for-profit businesses. The associations are many and factional, national and state, falling ideologically mainly along the lines of schism within chiropractic. Until relatively recently, the journals have not been peer-reviewed or indexed and the subluxation/vitalism-oriented ones still are not peer-reviewed in any meaningful sense of the term, nor are they indexed outside chiropractic circles [184, 185]. Research institutes have often been agenda-driven [186, 187] or simply practice building systems, and chiropractic diagnostic tools are incapable of detecting the lesions they claim to [183, 188].

Historically, organized medicine has used vitalism within chiropractic as a lever against legitimation. In 1963, the American Medical Association (AMA) formed a Committee on Quackery to “[determine] the true nature of chiropractic and its practitioners, and to inform the medical profession and the public of its findings” [189] p3. From this beginning, the American Medical Association mounted prolonged campaign to contain and eliminate the chiropractic profession as a health hazard in the United States. The AMA based its information campaign on two arguments:

1. The fundamental vitalistic tenets of chiropractic are pseudoscientific, untestable hypotheses in conflict with accepted scientific evidence.
2. The exaggerated, unsubstantiated claims by chiropractors about their care places the profession on a collision course with orthodox medicine and may be unsafe for the general public [190].

One of the AMA’s publications: *Chiropractic: The Unscientific Cult* advises

Either the theories and practices of scientific medicine are right and those of the cultists are wrong, or the theories and practices of the cultists are right and those of scientific medicine are wrong [189] p3.

The pamphlet included reproductions of advertisements claiming cures for diseases like cancer and mental illness. Chiropractors were quoted making statements against the effectiveness of vaccinations. Rather than science and evidence, the chiropractors cited in the pamphlet invoked an epistemology of appeal to authority; the “authority” was usually either D.D. or B.J. Palmer. The pamphlet also denigrated chiropractic teaching methods and the qualifications of the teachers themselves, both of which genuinely were inferior to those of medical schools [189] p9–12. In 1967, H. Doyl Taylor, secretary of the Committee on Quackery and a leading figure in AMA efforts on chiropractic, spoke at a “quackery workshop” held at Ball State University, framing the discussion with this statement: “As you know, [chiropractic] is a cult, about as far removed from scientific medicine, the diagnosis and treatment of human illness as it is possible to get” [191]. Unsubstantiated claims by the chiropractic profession [192–194] de-legitimises the profession by providing evidence it is failing to fulfil its obligations under the social contract. Sociologists Yvonne Villanueva-Russell [111] and Susan Smith-Cunnien [112] asserted that by defining chiropractic as deviant and using derogatory terms like unscientific cult, the AMA could frame itself as mainstream, reasonable, and scientific for social and political benefit.

The lack of legitimacy because of chiropractic adherence to a vitalistic ideology has had real effects on chiropractic around the world. One example is the failed attempt at starting a chiropractic course at Florida State University in 2005. In the USA, this would have been only the second university-based program and the first at a public university, and therefore a real achievement for the profession. According to the Sarasota Herald-Tribune: “The project has been ridiculed by FSU’s faculty, who say chiropractic medicine is a pseudoscience” [195]. In 2017 the Australian government undertook a review of Medicare reimbursement across the range of health care services. They noticed an anomaly in the ordering of multi-region spinal radiographs. Three and four-region spinal images were almost exclusively ordered by chiropractors (98.6%), so, in a clear example of loss of chiropractic’s professional autonomy, in 2017 Medicare rescinded reimbursement only to chiropractors for these x-ray series, stating:

To ensure whole spine X-rays are being used appropriately and safely, the Government is removing the ability of chiropractors to request whole spine X-rays… Patients will continue to be able to access spinal x-rays from other health practitioners where they are considered to be clinically appropriate, such as for assessing scoliosis [196].

Multi-region spinal radiographs are often ordered by chiropractors as part of many technique systems that require them for subluxation identification [197]. The practices of these practitioners de-legitimised the entire profession from the perspective of the Australian government, which acted to protect the public and reduce expenditure on procedures not supported by evidence. We conclude that vitalism in chiropractic is a barrier to legitimation.

#### Effect of vitalism on public acceptance

There is evidence that vitalism has either no effect or a negative effect on public acceptance of chiropractic. One study found that patients did not care about a chiropractor’s philosophy [ideology] or belief system, but just wanted someone to help with their pain. The theory behind treatment meant little to these patients; results meant everything [198]. Mootz [199] viewed subluxation theory as a useful way to explain some of the empirical clinical observations that chiropractic patients have reported but noted that justification for vitalistic beliefs regarding chiropractic effects on health can be confusing to the public and diminish professional credibility. He also indicated that it was not a patient-centred approach which is requisite within the twenty-first century health care system [199]. Denmark, Switzerland, and Alberta, Canada, show the highest utilization rates for chiropractic around the world, about twice that of other countries [200–203]. In these jurisdictions, chiropractors have largely abandoned traditional chiropractic ideology and embraced manual therapy for musculoskeletal issues. This would seem to indicate that vitalism is unnecessary for public acceptance and is likely a hindrance to it.

#### Effect of vitalism on integration

The places where chiropractic has had the best success with integration into government provision of health care are, not coincidentally, those places in which chiropractic has let go of its vitalistic roots and embraced modern musculoskeletal health care: Denmark, Switzerland, and Canada. Governments are always interested in providing service for the lowest cost. The first government investigation of cost-effectiveness for chiropractic was a study in Canada on low-back pain in 1993; it reported positive findings [204]. However, evidence for improvement in patient outcomes from the correction of chiropractic subluxations or postural changes has never been documented in a peer-reviewed, indexed journal. In 1972, chiropractic gained entry to the American Medicare system, based on a subluxation model. However, it was despite this model, not because of it, that chiropractors succeeded [205]. The public relations campaign taken up by the professional associations championed freedom, a powerful idea in American politics, and also resulted in at least a million letters being sent to politicians by chiropractors and their patients, [206] although one source said 3 million [207] and another 12 million [208]. This event influenced contemporaneous registration and government inquiries on chiropractic in several countries, but none adopted relief of the chiropractic subluxation as a basis for reimbursement [209–211]. In fact, continued insistence by vitalistic subluxation-based chiropractic prevented Medicare coverage of chiropractic in Australia even though chiropractic enjoyed significant public acceptance and was considered cost-effective [212]. The 1986 Medicare Benefits Review Committee [212] noted:

The continued claim by chiropractors to be able to treat “'Type 0" [organic or visceral] conditions is a major obstacle to us making any recommendations for public funding of chiropractic services in general. The adverse comments made about these treatments in the Webb Committee report and the Report of the New Zealand Commission of Inquiry remain valid today. p. 159

Chiropractor Michael Menke identified the focus on subluxation by some chiropractors, particularly to the exclusion of other outcome measures of health, as a barrier to integration [213]. Attorney and long-time chiropractic observer David Chapman-Smith also noted the danger of neglecting symptoms in chiropractic systems that focus on the supremacy of the subluxation in the role of disease [214]. Vitalist chiropractors shun diagnosis in favour of “analysis” of subluxations, a concept originally devised as a legal strategy to defend chiropractors accused of practising medicine without a license. Medical doctors diagnosed; chiropractors analysed; therefore chiropractors were not practising medicine [215, 216]. However, some have argued that the main strength for chiropractors in integrated health care practices is their diagnostic capabilities [213]. Musculoskeletal orthopaedics and neurological diagnosis is crucial to chiropractic being accepted into multidisciplinary situations, therefore vitalistic practitioners, focusing solely on the identification and correction of minor vertebral displacements are incongruent with the needs of these practices [217, 218]. Thus, vitalism is a barrier to chiropractic integration into health care systems.

#### Effect of vitalism on cultural authority

The term cultural authority, introduced by Paul Starr in 1982, denotes prominence and respect in a society resulting in professional monopoly and autonomy over its domain [109]. For this paper, we consider cultural authority to mean for chiropractic a high level of awareness by the community, credibility, esteem, and influence in strategic health care decision-making in public and private sectors. Even though public awareness of chiropractic is fairly good [219, 220], the profession has low cultural authority [221]. We attribute this to failing to achieve full professional status, poor legitimation, public acceptance, and integration. This cannot be due to the evidence-based musculoskeletal practitioners who share an understanding of the aetiology of pathology, use a common language with mainstream medical practitioners and are accepted onto health care teams. Therefore, this failure must be due to the continued presence of vitalism in chiropractic, with its alternative, and now discredited, paradigm of health and disease and its unique lexicon for expressing these concepts.

### The polarizing effect of modernization

Vriens, Vosselman, et al. note that over the past 30 years, professionals have been held to increasingly stringent standards [222]. This change has come about from a variety of pressures, including public scandals, incidents of malpractice, increased managerialism, and market competition coupled with increasing societal expectations. It is influencing professionals to work according to procedures and defined standards [222]. This is true for chiropractic, and additional pressure comes from the rise of evidence-based practice. Increasingly, health care providers are expected to demonstrate results from their efforts and to use methods that comport with a scientific understanding of health and disease. This is evident in the accreditation and registration standards wherever chiropractic is officially sanctioned by government registration. Furthermore, fiduciary law requires a health care provider to do more than just meet the standard of care within accreditation and registration standards [223].

The difficulty for chiropractic is that it is essentially two disparate groups held together by a common early history and name. As evidence-based practice continues to expand and strengthen, the groups can be seen to be increasingly divergent and the differences may be irreconcilable [10, 224].

The stress from being required to provide evidence for the clinical effectiveness of diagnostic and treatment methods seems to have a polarizing and galvanizing effect on chiropractors. Rather than adapting the paradigm of health to accommodate scientific advancement, vitalistic chiropractors seem to be becoming increasingly militant, levelling vitriolic ad hominem attacks on heretics and using emotion-laden labels as epithets, such as subluxation denier. Authors and institutions challenging vitalistic chiropractic are publicly labelled with the term [225–227]. In current times, using the word denier is usually aimed at people who are ignoring evidence about a certain topic, for instance, climate change deniers, evolution deniers, vaccine deniers [228]. However, with chiropractic the use is turned on its head, with the denier label being used on those who argue that vitalism and subluxation-based practice have little or no evidence to support them. Chiropractors tightly tied to their professional ideology might choose such a term to evoke negative associations to belittle their enemies. This idea is reinforced by highly emotive vitalist blog headlines such as: “Canadian Memorial Chiropractic College Joins Anti-Subluxation Hate Group” [133]. This vitriol in the defense of vitalism does not contribute to the professionalization of chiropractic.

## Conclusion

We assert that until chiropractic abandons the outdated concept of vitalism, it will never become a genuine mainstream status health care profession, at least by a definition that includes the moral and legal fiduciary duties to patients, all of which are necessary for the profession to uphold its social contract requirements. So long as a vitalistic ideology remains within chiropractic, it will remain separate and distinct, on the fringe of health care, an easy target for legitimate criticism from organized medicine, and therefore vulnerable to further marginalization by government regulation and private reimbursement services.

Even though chiropractic displays many of the attributes of a profession, legitimacy and cultural authority will remain out of reach as long as there is no consistent, coherent and defensible professional identity that comports with generally accepted concepts of disease and health and uses a language common with other health care providers. Abbott [149] provides advice that chiropractic would do well to note “societies have little time for experts who lack cultural legitimacy, irrespective of their success rate.” p. 54.

## Data Availability

Not applicable.

## Abbreviations

ACA: American Chiropractic Association
AMA: American Medical Association
CAM: Complementary and alternative medicine
HCP: Health care professional
ICA: International Chiropractors Association
ICEC: International Chiropractic Education Collaboration

## Footnotes

1 Where possible reference is made to publications from the late 1800s and early 1900s. This was the time when the topics of vitalism and mechanism were enjoying significant scrutiny within the scientific community. The papers selected were often seminal papers on the topic and hence it is worthwhile citing these interesting and informative authors.

2 “The Vienna Circle was a group of early twentieth-century philosophers who sought to reconceptualize empiricism by means of their interpretation of then recent advances in the physical and formal sciences. Their radically anti-metaphysical stance was supported by an empiricist criterion of meaning and a broadly logicist conception of mathematics. They denied that any principle or claim was to be accepted a priori.” The Vienna Circle posited a doctrine of unified science. Thus, no fundamental differences were seen to exist between the physical and the biological sciences or between the natural and the social sciences. http://plato.stanford.edu/archives/spr2016/entries/vienna-circle/
